# To Explore MR Imaging Radiomics for the Differentiation of Orbital Lymphoma and IgG4-Related Ophthalmic Disease

**DOI:** 10.1155/2021/6668510

**Published:** 2021-02-04

**Authors:** Ye Yuan, Guangyu Chu, Tingting Gong, Lianze Du, Lizhi Xie, Qinghai Yuan, Qinghe Han

**Affiliations:** ^1^Department of Radiology, The Second Hospital of Jilin University, Changchun 130041, China; ^2^GE Healthcare, MR Research China, Beijing 100176, China

## Abstract

Among orbital lymphoproliferative disorders, about 55% of diagnosed cancerous tumors are orbital lymphomas, and nearly 50% of benign cases are immunoglobulin G4-related ophthalmic disease (IgG4-ROD). However, due to nonspecific characteristics, the differentiation of the two diseases is challenging. In this study, conventional magnetic resonance imaging-based radiomics approaches were explored for clinical recognition of orbital lymphomas and IgG4-ROD. We investigated the value of radiomics features of axial T1- (T1WI-) and T2-weighted (T2WI), contrast-enhanced T1WI in axial (CE-T1WI) and coronal (CE-T1WI-cor) planes, and 78 patients (orbital lymphoma, 36; IgG4-ROD, 42) were retrospectively reviewed. The mass lesions were manually annotated and represented with 99 features. The performance of elastic net-based radiomics models using single or multiple modalities with or without feature selection was compared. The demographic features showed orbital lymphoma patients were significantly older than IgG4-ROD patients (*p* < 0.01), and most of the patients were male (72% in the orbital lymphoma group vs. 23% in the IgG4-ROD group; *p* = 0.03). The MR imaging findings revealed orbital lymphomas were mostly unilateral (81%, *p* = 0.02) and wrapped eyeballs or optic nerves frequently (78%, *p* = 0.02). In addition, orbital lymphomas showed isointense in T1WI (100%, *p* < 0.01), and IgG4-ROD was isointense (60%, *p* < 0.01) or hyperintense (40%, *p* < 0.01) in T1WI with well-defined shape (64%, *p* < 0.01). The experimental comparison indicated that using CE-T1WI radiomics features achieved superior results, and the features in combination with CE-T1WI-cor features and the feature preselection method could further improve the classification performance. In conclusion, this study comparatively analyzed orbital lymphoma and IgG4-ROD from demographic features, MR imaging findings, and radiomics features. It might deepen our understanding and benefit disease management.

## 1. Introduction

Orbital lymphoproliferative disorders (OLPDs) consist of a broad range of benign and malignant tumors [1, 2]. Among diagnosed cancerous tumors, nearly 55% of cases are orbital lymphomas [3], while luckily, most orbital lymphomas are primary, low-grade, and amendable to low-dose radiotherapy [1–3]. To improve the diagnosis performance, many studies explored to figure out some discriminative characteristics. Eckardt et al. evaluated the diagnostic approach in 11 orbital lymphoma patients and found that orbital swelling, pain, and motility impairment were the leading clinical symptoms [4]. Another study observed the proptosis, eyelid lesions, decreased visual acuity, and optic nerve compression in 26 cases with orbital lymphoma [5]. Moreover, Priego et al. described different orbital lymphoma patterns at diagnosis and follow-up in 19 cases, and superior-lateral quadrant and extraconal location were predominantly observed on imaging scans [6]. The patterns were further confirmed by Jin et al. who evaluated the computed tomography (CT) imaging and magnetic resonance imaging (MRI) features of primary orbital lymphoma to establish a differential diagnosis in 14 cases, reporting that periorbital preseptal tissues were mainly involved in the upper lateral quadrant of the orbit [7]. They also suggested that MRI may be very useful for assessing the location, configuration, inner structure, and characteristic manifestations of orbital lymphomas [7]. However, these symptoms were either qualitative such as laterality or nonspecific such as decreased visual acuity; thus, they could not have a wider application [1–8].

It is also figured out that nearly 50% of benign OLPD cases are immunoglobulin G4-related ophthalmic disease (IgG4-ROD) [1, 9]. IgG4-ROD is an inflammatory disease of unknown etiology, which can be treated using corticosteroid therapy [1, 2]. Typical IgG4-ROD is characterized by painless enlarging masses over the lacrimal gland with or without proptosis. Bilateral disease is common but not necessarily symmetrical; visual acuity is usually not impaired. Besides the lacrimal gland, IgG4-ROD has been reported in various orbit tissues, including muscle, fat, eyelid, and nerve [9]. A short summary of related publications indicated that the signs and symptoms of IgG4-ROD included chronic noninflammatory lid swelling and proptosis. Moreover, patients often had a history of allergic disease and increased serum levels of IgG4, IgE, and hypergammaglobulinemia [10]. In addition, a study comparing both IgG4-ROD and non-IgG4-ROD European patients revealed that infraorbital nerve enlargement was frequently presented in IgG4-ROD patients [11].

Except for conventional MR images, diffusion-weighted imaging (DWI) has been extensively explored over the years. The apparent diffusion coefficient (ADC) values have been revealed useful in diagnosing OLPDs [12, 13]. Haradome et al. observed that the mean ADC of orbital lymphomas was significantly lower than that of benign OLPDs (*p* < 0.01). In addition, an optimal cutoff of ADC values could yield a superior prediction of orbital lymphoma, and the prediction was even better than that using the contrast-enhancement ratio of lesions [1]. Xu et al. also found significantly lower ADC (*p* < 0.001) in malignant OLPDs when compared to benign ones, and a receiver operating characteristic curve analysis indicated ADC alone could achieve an optimal sensitivity in the classification of benign and malignant OLPDs [2]. In addition, ElKhamary et al. suggested that median ADC was significantly different between benign and malignant OLPDs, and an ideal threshold of ADC values benefited the classification of diffuse orbital masses [14]. Notably, Lecler et al. [15] and Maldonado et al. [16] also reported similar results.

CT is another useful imaging approach for analyzing OLPDs. Jin et al. [7] found that isodense soft tissue masses characterized primary orbital lymphoma with clear demarcation on CT images; the lesions showed homogeneously marked enhancement when contrast medium was used. Simon et al. [17] discovered that benign lesions were more likely hyperdense or hypodense on CT in comparison with inflammatory and malignant tumors. Briscoe et al. [5] suggested that bone changes were more common on CT images when orbital lymphomas were suspected. Thus, combining CT and MR imaging could be useful for accurate diagnosis of OLPDs.

Preoperative identification of orbital lymphoma and IgG4-ROD facilitates disease management, treatment planning, and health care [1–3]. Yet, due to nonspecific presenting signs and symptoms and lack of qualitative findings, diagnosis is still somehow challenging. For diagnosis, a biopsy is routinely utilized in clinics. However, considering the tumor's specific location, i.e., orbital lesions, a biopsy is difficult, and thus, may lead to misdiagnosis, mistreatment, and even missed diagnosis [9].

Radiomics has been widely explored for intelligent diagnosis [18–20]. It extracts quantitative features from medical images using advanced algorithms [21–23], and the features are further mined for disease diagnosis and cancer staging [24–27]. However, to the best of our knowledge, no machine learning-based radiomics models have yet been designed for orbital lymphoma and IgG4-ROD. Since previous studies suggested that MR imaging is a promising tool to accurately visualize the location, shape, and internal structure of orbital lymphoma [1, 2, 7, 11]; in this study, we assessed the value of conventional MR images in machine learning-based radiomics approaches for clinical identification of orbital lymphoma and IgG4-ROD.

## 2. Materials and Methods

### 2.1. Patients and Data Collection

This retrospective study was approved by the institutional review board of the Second Hospital of Jilin University, and written informed consent from patients was waived. Through a review of our hospital database, 36 cases of orbital lymphoma and 42 cases of IgG4-ROD were identified. All patients were historically confirmed by surgical biopsy between March 2013 and September 2018. It should be noted that all patients received MR imaging before the surgical biopsy.

Histopathologic features were used for pathologic diagnosis. Orbital lymphoma was diagnosed using flow cytometric and gene rearrangement analysis. IgG4-ROD was identified according to the immunohistochemical staining results, which require the number of IgG4-positive plasma cells more than 50 cells per high-power field samples, the ratio of IgG4-positive plasma cells over IgG-positive plasma cells >40%, and serum IgG4 concentration of 1.35 g/L [28].

All diagnosed patients were without a history of previous treatment or surgery. They had no history of orbital diseases or other tumors. All imaging was performed using a 3.0-T MR equipment (GE MR 750) with imaging parameters as in Table 1. Axial fast spin-echo (FSE) T1-weighted (T1WI) and T2-weighted (T2WI) images, contrast-enhanced T1WI in the axial (CE-T1WI) and coronal (CE-T1WI-cor) planes were acquired using Gd-DTPA (dose: 0.1 mmol/kg; and injection rate: 2.0 ml/s).

### 2.2. Manual Annotation and Feature Extraction

Mass lesions were manually outlined by using the ITK-SNAP software (version 3.8.0). Two board-certified radiologists with 6 and 10 years of experience in head and neck imaging performed the manual annotation together and were blinded to clinical information and histologic diagnosis. If consensus was not reached, the annotation was further arbitrated by a senior radiologist with 16 years of experience to ensure the annotation quality.

Manual annotation and feature extraction were performed as follows: MR images of one patient were imported into the ITK-SNAP. Then, the radiologists performed the image analysis from the laterality (left/right/bilateral) and the shape of the margins (well-defined or ill-defined) to figure out obvious lesion boundaries. If the lesion boundaries were ambiguous, MR images from the four imaging sequences were displayed for observation, and CE-T1WI and CE-T1WI-cor were set as the baseline. After discussion, the consensus was reached, and lesion delineation was performed slice by slice. Specifically, the delineation was made from the head to the feet direction to avoid bone structures and eyeball regions. When eye muscles and/or optic nerves were involved, eye muscles and organ tissues were outlined if necessary, as the lesion was our point of interest.

**Figure 1:**
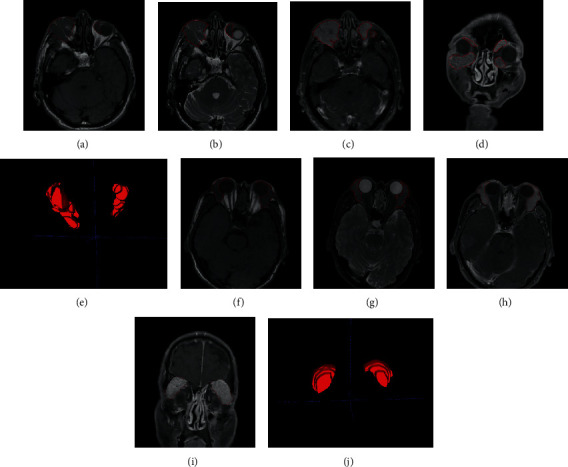
Two representative cases. The top row shows a 60-year male patient with orbital lymphoma, and the bottom row shows a 60-year female patient with IgG4-ROD. In each case, one image of T1WI, T2WI, CE-T1WI, and CE-T1WI-cor and the volume mask are shown from left to right. Mass lesions are the region in red lines. Note that images are cropped for display purposes.

Two representative examples are shown in Figure 1. The top row represents a case of orbital lymphoma, and the bottom row shows a case of IgG4-ROD. From left to right is one slice of T1W1, T2W1, CE-T1WI, and CE-T1WI-cor image in addition to the mask of volume region of interest. In each slice, the region in red lines represents the mass lesion.

Annotated tumors were quantified using a public package Pyradiomics (version 3.0), and a total of 99 features were computed. Among the features, 14 were for shape description, 18 were from first-order histogram analysis, 22 were from gray-level cooccurrence matrix (GLCM) analysis, 14 were from gray-level run-length matrix (GLRLM) features, 16 were from gray-level size zone matrix (GLSZM) analysis, and 15 were from gray-level differential matrix (GLDM) analysis. These features have been applied for lesion representation, radiomics, and intelligent diagnosis [29].

### 2.3. Disease Differentiation

**Figure 2:**
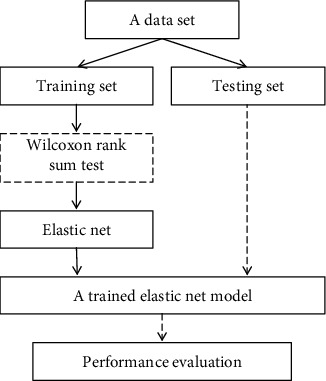
The procedure of disease diagnosis. It includes data splitting, identification of significant features, elastic net-based feature selection, disease diagnosis, and performance assessment.

Figure 2 shows the workflow of disease differentiation using elastic net fitting [30]. First, a data set was divided into a training set and a testing set by random splitting. The Wilcoxon rank-sum test was optionally used to figure out these statistically significant features by comparing the two groups of data samples. Consequently, the default parameters of the elastic net were tuned, finally generating a trained model. At the testing stage, the trained elastic net was evaluated via a testing data set, and its performance was assessed. The rectangle with a dashed line indicated a comparison study to investigate the effect of the Wilcoxon rank-sum test in disease diagnosis.

### 2.4. Experimental Design

This study investigated the effect of single modality, multiple modalities, and preselection of important features on disease classification performance. Single modality data sets included T1WI, T2WI, CE-T1WI, and CE-T1WI-cor; multiple modality data sets comprise different combinations of single modality data (T1WI + T2WI + CE-T1WI, T1WI + T2WI + CE-T1WI-cor, CE-T1WI + CE-T1WI-cor, and T1WI + T2WI + CE-T1WI + CE-T1WI-cor). In addition, the effect of selecting statistically important features using a nonparametric test of Wilcoxon rank-sum test on disease diagnosis was observed.

The elastic net has been widely used in feature selection, regularized regression, and data classification [30]. It linearly combined both *L*_1_ and *L*_2_ penalties using a parameter *α* to overcome some limitations of the least absolute shrinkage and selection operator (LASSO) [31]. In this study, the elastic net was used for feature selection and classification (*α* = 0.75). First, 80% of data samples were randomly selected for training the elastic net model, and 10-folder cross-validation was used for automatic parameter tuning. Next, the trained elastic net model was tested on the testing samples. Then, the prediction performance was evaluated using four metrics, including the area under the curve (AUC), accuracy (ACC), sensitivity (SEN), and specificity (SPE) [32]. In addition, the procedure was repeated 100 times, and the performance metrics were averaged. The whole procedure was implemented with MATLAB2018a (MathWorks, USA) and the elastic net using the embedded function “lasso.m.”

### 2.5. Statistical Analysis

The group differences were assessed by a two-tailed *t*-test or Pearson's chi-squared test based on the SPSS software (version 25.0, IBM Corp., Armonk, NY). *p* value <0.05 was considered statistically significant.

## 3. Results

### 3.1. Patient Characteristics and Tumor Distribution

Table 2 shows patient characteristics and tumor distribution between the two groups. Significant differences were found between groups. Patients with orbital lymphoma were 9 years older than patients with IgG4-ROD. Moreover, most patients with malignant tumors were male (26/36, 72%). In the IgG4-ROD group, 10/42 (23%) were male. Yet, no statistical difference in gender was found between the two groups. In addition, most orbital lymphomas were unilaterally involved (29/36, 81%), while IgG4-RODs were equally unilateral and bilateral.

Table 3 summarizes MR features between the two groups. Significant differences were found in 3 attributes. First, the shape of margins of IgG4-ROD lesions was well-defined (27/42, 64%) in comparison with that of orbital lymphomas (14/36, 39%). Second, the lesions were more frequently wrapped around eyeballs and/or optic nerves in patients with orbital lymphomas (28/36 (78%)) compared to those with IgG4-RODs (22/42 (52%)). Third, in T1WI images, orbital lymphoma was perceived as isointense (36/36, 100%), while IgG4-ROD as isointense (25/42, 60%) or hyperintense (17/42, 40%). We also found that some patients in both groups had flow void sign and in-homogeneity in lesion regions. Moreover, most orbital lymphomas (26/36, 72%) and IgG4-RODs (31/42, 74%) were perceived as hypointense signals in T2WI images.

### 3.2. Parameter Optimization

**Figure 3:**
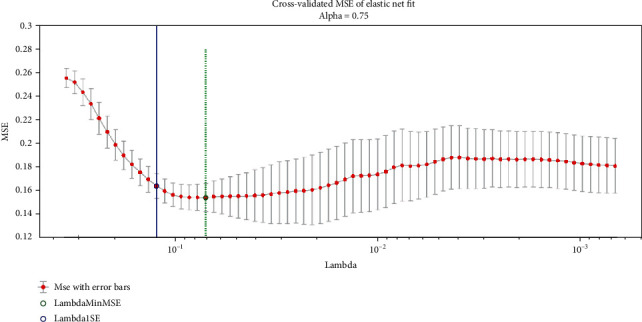
Automated optimization of the parameter *λ* when the training samples are fitted by elastic net using 10-folder cross-validation (CV). The *x*-axis shows the change of *λ*, and the *y*-axis indicates the mean square error (MSE). The green dotted line locates the *λ* with minimum CV error, and the solid blue line points to the minimum CV error plus one standard deviation (SE).

Figure 3 shows the automated optimization *λ* when training samples were fitted by elastic net using 10-folder cross-validation (CV). The *x*-axis indicates the change of *λ* value, and the *y*-axis corresponds to the mean square error (MSE). In addition, the green dotted line locates the *λ* with minimum CV error, and the solid blue line points to the minimum CV error plus one standard deviation (SE). In this study, a larger *λ* was used when the MSE was within one SE of the minimum one for the consideration of model reliability.

### 3.3. Performance Using Single versus Multiple Modality Data

Table 4 summarizes the performance when using single or multiple modality data for disease classification. The best result was obtained when using CE-T1WI, followed by CE-T1WI-cor. Both T1WI and T2WI caused poor SPE (<0.50), while T1WI led to a fair AUC value (<0.60). The application of multimodality increased the diagnosis results. The addition of CE-T1WI-cor increased the AUC and SPE by 5% and 9%, respectively. However, adding T1WI and T2WI to the combination of CE-T1WI + CE-T1WI-cor did not improve the classification performance.

### 3.4. Performance with Feature Preselection

The results with feature preselection are shown in Table 4. By comparing both Table 4 and Table 5, we found that feature preselection improved the combination of CE-T1WI and CE-T1WI-cor (*p* < 0.02) and benefited single- (such as CE-T1WI and CE-T1WI-cor) and other multiple modality data (such as T1WI + T2WI + CE-T1WI) based disease differentiation.

### 3.5. Feature Analysis

Wilcoxon rank-sum test indicated that 13, 18, 75, and 40 features were with statistical significance (*p* < 0.05) corresponding to T1WI, T2WI, CE-T1WI, and CE-T1WI-cor. In disease classification, elastic net further verified that 1, 5, 6, and 4 features were frequently selected (>50 times) between the two groups of patients on T1WI, T2WI, CE-T1WI, and CE-T2WI-cor, respectively. It is worth noting the elastic net model with the superior performance required 6 features, among which 5 were computed from CE-T1WI (1 shape feature, major axis length; 2 GLCM features, correlation and autocorrelation; 1 GLDM feature, large dependence high gray-level emphasis; 1 GLRM feature, long-run high gray-level emphasis), and 1 GLDM feature (large dependence high gray-level emphasis) from CE-T2WI-cor. In addition, frequently selected features were all from postcontrast T1WI images, and the GLDM feature was highlighted.

## 4. Discussion

This study investigated demographic characteristics, MR imaging features, and radiomics models of orbital lymphoma and IgG4-ROD, thus aiming to facilitate preoperative diagnosis of these two different tumor types. Seventy-eight patients were retrospectively reviewed, and mass lesions were manually annotated. Clinical characteristics, MR findings, and the performance of single and multimodality data with and without feature preselection were analyzed.

Demographic characteristics revealed that orbital lymphoma patients were significantly older than IgG4-ROD patients. This has also been previously reported by other studies that examined the difference between orbital lymphoma and other diseases, such as benign OLPDs [1, 2], pseudotumor [33], and lymphoma subtypes [34]. Thus, the patient's age should be considered when performing a diagnosis. Moreover, we discovered that most patients with orbital lymphoma (72%) were male, yet there was no significant difference between patients with orbital lymphoma and those with IgG4-ROD, which is consistent with data published by Olsen and Steffen [34] and inconsistent with some other studies [1, 2, 33]. Therefore, the predominance of male patients in orbital lymphoma requires to be further investigated by future clinical studies.

MR imaging features suggested orbital lymphomas had unilateral involvement compared to benign OLPDs, which was consistent with previous data [1, 2, 6–8, 33, 34]. Moreover, orbital lymphomas were frequently located around organs, such as eyeballs, and compress optic nerves, which might explain the decreased visual acuity, eye irritation, excessive tearing, and pain in the eye in these patients [6, 8, 34]. In addition, when comparing the signal intensity with that of the cerebral cortex, orbital lymphomas showed isointense in T1WI and hypointense signals in T2WI. At the same time, IgG4-RODs had iso- or hyperintense signals in T1WI and hypointense signals in T2WI. As to the shape of margins, most IgG4-RODs were well-defined, which was verified by prior disease classification [7]. However, these MR findings, nonspecific, qualitative, and subjective, could be found between orbital lymphoma and other non-IgG4-ROD. Thus, these nondiscriminative features might require other advanced imaging modalities for deeper understanding.

Experimental results highlighted the importance of CE-T1WI for disease classification. CE-T1WI achieves superior performance, and in combination with CE-T1WI-cor and preselection of features, it could further improve the diagnostic performance. Contrast-enhanced T1-weighted MR imaging was highlighted in this study. Six discriminative features (5 from CE-T1WI and 1 from CE-T1WI-cor) were retrieved. As these features were quantitative and meaningful, they can help understand the machine learning-based radiomics models. On the other hand, two studies explored machine learning methods for the quantitative analysis of ocular adnexal lymphoma and idiopathic orbital inflammation. Guo et al. discovered that five features (4 from CE-T1WI and 1 from T2WI) achieved a larger AUC (> 0.70) [35]. Hou and his colleagues found bag-of-words features from CE-T1WI may significantly outperform the features from no-enhanced MR images [36]. In general, both studies indirectly provided support for our findings, suggesting that contrast-enhanced MR imaging may improve the differentiation between orbital lymphoma and IgG4-ROD.

To our knowledge, this is the first study that aimed at building a machine learning model for the differentiation of orbital lymphoma and IgG4-ROD. The elastic net is the backbone of the proposed radiomics model. It retrieves informative features for data representation and also acts as the classifier for disease prediction. When analyzing the performance of single- and multimodal data, CE-T1WI resulted as the most informative. To reduce the feature number, improve the prediction performance, and enhance the model interpretability, feature preselection via statistical comparison was conducted, and a handful of features were identified. The present study proposed a radiomics model, which revealed the importance of CE-T1WI in the classification and might further be used to screen and diagnose eye diseases.

This study also has a few limitations. First, T1WI, T2WI, and CE-T1WI are conventional MR imaging modalities, yet other modalities, such as DWI and CT, and some other parameters, such as contrast-enhancement ratio and ADC, should also be considered. Second, a limited number of features were collected for tumor description; more features should be collected to quantify mass lesions from various perspectives. Third, this study applied elastic net for feature selection and disease diagnosis. Several other approaches, such as feature ranking methods [37], can be used for feature selection in this binary classification task. Finally, the sample size was small, and large-scale studies are required to confirm these findings.

## 5. Conclusion

In the present study, several quantitative MR features were identified as relevant for differentiation of orbital lymphoma and IgG4-ROD. The machine learning-based radiomics model verified that contrast-enhanced T1 MR imaging was discriminative in disease classification. The next step is to incorporate other modalities and advanced techniques to further explore the differences between diseases.

## Figures and Tables

**Table 1:** MR imaging parameters on the 3.0-T scanner.

	TR (ms)	TE (ms)	Slice thickness (mm)	Slice gap (mm)	Matrix size	Field of view ([mm, mm])
T1W1	515	17	3.0	0.3	[512, 512]	[15, 15]
T2WI	2000	85	3.0	0.3	[512, 512]	[15, 15]
CE-T1WI	463	8.5	3.0	0.3	[512, 512]	[15, 15]
CE-T1WI-cor	650	8.8	3.0	0.3	[512, 512]	[15, 15]

**Table 2:** Patient characteristics and tumor distribution.

	Orbital lymphoma (*n* = 36)	IgG4-ROD (*n* = 42)	*p* value	*K* value
Age (years)			< 0.01	
Mean ± std	64.89 ± 10.30	55.21 ± 13.88		
Range	[38, 84]	[25, 78]		
Gender			0.03	4.85
Male	26	20		
Female	10	22		
Laterality			0.02	7.53
Left	18	11		
Right	11	11		
Bilateral	7	20		

**Table 3:** Perceived MR imaging features.

	Orbital lymphoma (*n* = 36)	IgG4-ROD (*n* = 42)	*p* value	*K* value
Margin			<0.01	37.05
Well-defined	14	27		
Ill-defined	22	15		
Local spread of eyeball or optic nerve			0.02	5.43
Yes	28	22		
No	8	20		
Extraocular muscles involved			0.12	2.47
Yes	15	25		
No	21	17		
Flow void sign present on T2WI			0.23	1.44
Yes	14	11		
No	22	31		
Signal intensity on T1WI			<0.01	18.63
Low	0	0		
Iso	36	25		
High	0	17		
Signal intensity on T2WI			0.59	1.04
Low	26	31		
Iso	9	8		
High	1	3		
Homogeneity			0.75	0.10
Yes	29	35		
No	7	7		

**Table 4:** Disease classification using single or multiple modality data.

	AUC	ACC	SEN	SPE
T1WI	0.54 ± 0.10	0.53 ± 0.13	0.79 ± 0.16	0.29 ± 0.20
T2WI	0.63 ± 0.12	0.62 ± 0.13	0.79 ± 0.11	0.46 ± 0.18
CE-T1WI	0.74 ± 0.10	0.74 ± 0.11	0.81 ± 0.16	0.67 ± 0.16
CE-T1WI-cor	0.72 ± 0.10	0.72 ± 0.11	0.83 ± 0.14	0.61 ± 0.21
T1WI + T2WI + CE-T1WI	0.70 ± 0.12	0.70 ± 0.12	0.79 ± 0.10	0.62 ± 0.14
T1WI + T2WI + CE-T1WI-cor	0.71 ± 0.12	0.69 ± 0.14	0.85 ± 0.10	0.58 ± 0.16
T1WI + T2WI + CE-T1WI + CE-T1WI-cor	0.78 ± 0.10	0.77 ± 0.10	0.82 ± 0.14	0.74 ± 0.17
CE-T1WI + CE-T1WI-cor	0.79 ± 0.11	0.78 ± 0.11	0.82 ± 0.15	0.76 ± 0.19

**Table 5:** Disease classification with feature preselection.

Wilcoxon rank-sum test	AUC	ACC	SEN	SPE
T1WI	0.57 ± 0.09	0.55 ± 0.11	0.74 ± 0.16	0.39 ± 0.19
T2WI	0.59 ± 0.12	0.58 ± 0.13	0.80 ± 0.14	0.38 ± 0.18
CE-T1WI	0.73 ± 0.11	0.73 ± 0.11	0.78 ± 0.15	0.68 ± 0.19
CE-T1WI-cor	0.74 ± 0.10	0.73 ± 0.11	0.86 ± 0.12	0.61 ± 0.21
T1WI + T2WI + CE-T1WI	0.75 ± 0.11	0.74 ± 0.11	0.80 ± 0.11	0.69 ± 0.12
T1WI + T2WI + CE-T1WI-cor	0.70 ± 0.10	0.69 ± 0.12	0.83 ± 0.10	0.58 ± 0.16
T1WI + T2WI + CE-T1WI + CE-T1WI-cor	0.78 ± 0.11	0.78 ± 0.10	0.83 ± 0.13	0.73 ± 0.18
CE-T1WI + CE-T1WI-cor	0.82 ± 0.09	0.81 ± 0.09	0.84 ± 0.12	0.79 ± 0.14

## Data Availability

The MR images that support this study's findings are restricted by the Medical Ethics Committee of the Second Hospital of Jilin University to protect patient privacy. Requests for access to the data sets or the radiomics features can be made to the corresponding author Qinghe Han (hanqinghehe@126.com).
